# Health System Barriers to Access and Use of Magnesium Sulfate for Women with Severe Pre-Eclampsia and Eclampsia in Pakistan: Evidence for Policy and Practice

**DOI:** 10.1371/journal.pone.0059158

**Published:** 2013-03-26

**Authors:** Maryam Bigdeli, Shamsa Zafar, Hafeez Assad, Adbul Ghaffar

**Affiliations:** 1 Alliance for Health Policy and System Research, World Health Organization, Geneva, Switzerland; 2 Centre of Excellence in Maternal, Newborn and Child Health, Health Services Academy, Islamabad, Pakistan; 3 Health Services Academy, Islamabad, Pakistan; The University of Adelaide, Australia

## Abstract

Severe pre-eclampsia and eclampsia are rare but serious complications of pregnancy that threaten the lives of mothers during childbirth. Evidence supports the use of magnesium sulfate (MgSO4) as the first line treatment option for severe pre-eclampsia and eclampsia. Eclampsia is the third major cause of maternal mortality in Pakistan. As in many other Low- and Middle-Income Countries (LMIC), it is suspected that MgSO4 is critically under-utilized in the country. There is however a lack of information on context-specific health system barriers that prevent optimal use of this life-saving medicine in Pakistan. Combining quantitative and qualitative methods, namely policy document review, key informant interviews, focus group discussions and direct observation at health facility, we explored context-specific health system barriers and enablers that affect access and use of MgSO4 for severe pre-eclampsia and eclampsia in Pakistan. Our study finds that while international recommendations on MgSO4 have been adequately translated in national policies in Pakistan, the gap remains in implementation of national policies into practice. Barriers to access to and effective use of MgSO4 occur at health facility level where the medicine was not available and health staff was reluctant to use it. Low price of the medicine and the small market related to its narrow indications acted as disincentives for effective marketing. Results of our survey were further discussed in a multi-stakeholder round-table meeting and an action plan for increasing access to this life-saving medicine was identified.

## Introduction

Pre-eclampsia is a disorder associated with high blood pressure and proteinuria during pregnancy (1). Severe pre-eclampsia and eclampsia are rare but serious complications of pregnancy that threaten the lives of mothers during childbirth. It is estimated that 40,000 women die from these complications each year [1], mostly in Low- and Middle-Income countries (LMIC).

Evidence supports the use of magnesium sulfate (MgSO4) as the first line treatment option for severe pre-eclampsia and eclampsia [2], [3]. A recent systematic review of trials demonstrated that MgSO4 significantly reduces the risk of eclampsia by more than half with only minor reported side effects [4]. Magnesium Sulfate is listed as a priority medicine for mothers and children by the World Health Organization [5] and is part of the essential commodities required to deliver the package of interventions for achieving the Millenium Development Goal 5 (MDG5) [6], [7].

Despite clear evidence and recommendations of international organizations such as the World Health Organization, fact is remaining that access to and use of MgSO4 in LMIC is far from optimal. Evidence collected from maternity units of hospitals with more than 1000 deliveries in Mexico and Thailand shows that MgSO4 is underutilized in women with eclampsia [8]. Sevene *et al.* report on barriers to the use of this life-saving medicine in Mozambique and Zimbabwe [9] and identify a range of market and system failures that explain the situation: these include the low price of generic MgSO4 which is a deterrent to effective marketing practices by pharmaceutical companies and a lack of specific public intervention to correct the situation. Similarly, Ridge *et al*. identify enablers and barriers to access and use of MgSO4 in Zambia [10]. These barriers and enablers take place at policy, regulatory and implementation levels.

The Pakistan demographic and health survey of 2007 shows a high Maternal Mortality Ratio (MMR 278/100,000 live births) and the country lags behind in achieving MDG5 [11]. Eclampsia is the third major cause of maternal mortality in Pakistan. As in many other LMIC, it is suspected that MgSO4 is critically under-utilized in the country. There is however a lack of information on context-specific health system barriers that prevent optimal use of this life-saving medicine in Pakistan.

The objectives of this paper are therefore to identify health system barriers, both at policy and implementation levels, to access and use of MgSO4 in Pakistan; and to provide recommendations for removing these barriers through an evidence-based action plan.

## Methods

Ethical approval was obtained from the ethics review committee of Health Services Academy (HSA) in Islamabad and relevant permissions from the respective departments were sought. All participants in focus group discussions and face-to-face interviews participated in their professional capacity. A standard information sheet was used to inform them about the study objectives. Verbal consent was obtained from participants in focus group discussions to avoid disrupting the dynamic of the facilitated discussion, while written consent was obtained from all interviewees. For verbal consent procedure, the standard information sheet used to inform focus group discussion participants are kept and bear the signature of all participants on the sheet. A retrospective review of patients’ charts in visited facilities was originally planned following written consent from the patient or a family member. However, given the nature of the conditions studied, no hospitalized patient was found during the time of the visit. The consent procedure was approved by the ethics review board of HSA.

### 1. Conceptual Framework

**Figure 1:**
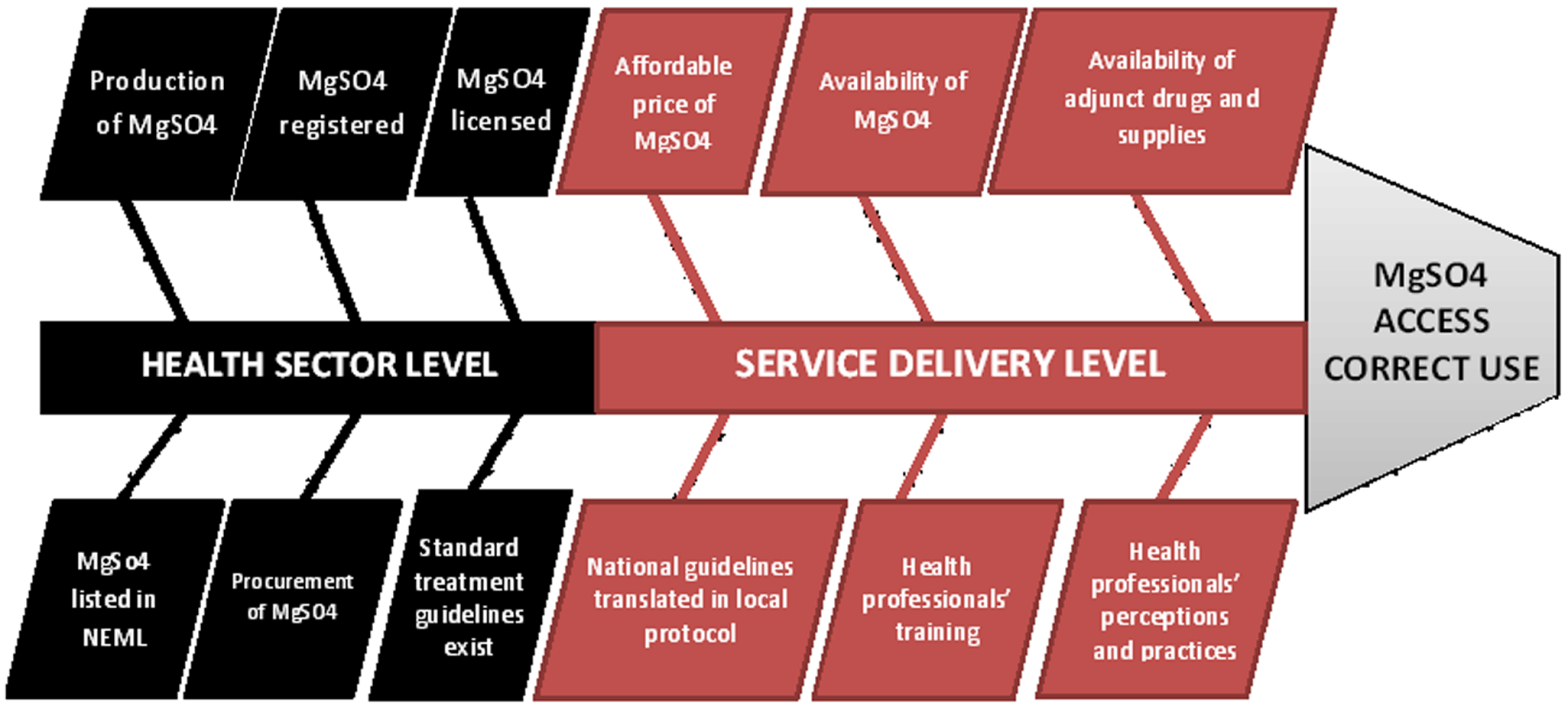
Fishbone Diagram: health system requirements for access to and use of MgSO4 for eclampsia and severe pre-eclampsia (adapted from Ridge et al).

Constraints to access to medicines occur at five levels of the health system: individual, households and communities; health service delivery; health sector; national level beyond the health sector and international level [12]. For this study, we concentrate on health sector and health service delivery levels. The Fishbone (or Ishikawa) Diagram developed by Ridge *et al.* [10] was used to map determinants of access to and use of MgSO4 at health sector and health service delivery levels. Ridge *et al.* identify the following requirements (or critical components) for adequate access to and use of MgSO4 in their Fishbone Diagram: inclusion of MgSO4 in National Essential Medicines List (NEML) and Standard Treatment Guidelines (STG), registration in the country for use in treatment of severe pre-eclampsia and eclampsia, presence of a suitable procurement and distribution system, presence of a suitable local protocol for use by health facilities providing basic and emergency obstetric care, awareness and adequate training of health professionals on the use of MgSO4 as first line treatment for severe pre-eclampsia and eclampsia, availability of supplies and equipment to administer MgSO4 at facility level. Ridge *et al.* also identify access to antenatal care services as a requirement to access to and use of MgSO4. After discussions with key stakeholders (policy makers, clinicians, pharmacists) in Pakistan, the list of requirements put forward by Ridge *et al.* was retained with the exception of ‘access to antenatal care services’, which is a requirement for access to any alternative treatment for eclampsia (MgSO4, diazepam or other) and likely to be a confounding factor. Affordable price of MgSO4 was added as a requirement. The modified Fishbone Diagram used in this study is presented in Figure 1.

### 2. Identification of Health System Barriers and Enablers

Based on the requirements presented in our Fishbone Diagram, we collected context-specific information on existence of health system barriers and enablers to access and use of MgSO4 in Pakistan. In our study, we add interviews and focus group discussions with health professionals and policy makers to data collection methods used by Ridge *et al.* [10]. Data collection tools were designed to collect information at each level of the health system, through a mixed methods approach combining the following: 1) desk review of relevant policy and regulatory documents, 2) in-depth interviews and focus group discussions with key stakeholders at national and provincial levels, and 3) facility survey including in-depth interviews of relevant health staff, review of charts and direct observation of obstetric and pharmacy wards.

A desk review of available evidence from published and grey literature, national documents such as NEML, STG, other clinical guidelines and national policies in vigour was undertaken. In-depth interviews were conducted with a total of 48 stakeholders using a semi-structured interview questionnaire. These stakeholders consisted in 5 senior government officials of the Ministry of Health (MoH) and Maternal, Newborn and Child Health Program (MNCH); 12 pharmacists including employees of the Central Medical Store (CMS), 18 doctors and 13 nurses or midwives. Data was collected on the policies and practices in place in Pakistan with regard licensing, registration, procurement, supply and use of MgSO4. Interviewees were also asked to comment on factors affecting the effective use of the drug in general and in their own facilities in particular. Focus group discussions were carried out with a total of 16 women medical officers and practicing obstetricians as well as professors from all provinces. The focus group discussions covered their knowledge and awareness about MgSO4, their knowledge of policies and practices in place in Pakistan, the challenges faced with implementation as well as their suggestions for improvement.

A purposive sample of 15 health facilities was surveyed in Baluchistan (n = 3), Khaibar Pakhtun Khawa (n = 2), Punjab (n = 6) and Sindh (n = 4). These included five teaching hospitals; seven non-teaching facilities providing comprehensive Emergency Obstetric and Neonatal Care (EmONC) among which two private structures; and three facilities providing basic EmONC. The pharmacy and obstetric departments of facilities were visited for data collection based on on-site observation, interviews and charts or files review.

On-site observation was undertaken to ascertain availability of a 24 hours supply of the drug for one patient. Moreover, the observation assessed whether the facility had the necessary supplies and equipment to administer MgSO4 injection and monitor the patient (calcium gluconate, lignocaine, sterile injection material). The route of administration and the challenges faced were discussed with the clinicians and pharmacists of the health facility. The presence of guidelines/protocols in the facility was also observed.
In-depth interviews with personnel of the obstetric ward (midwives, doctors and nurses) assessed their first line treatment choice for severe pre-eclampsia or eclampsia, the reasons for this choice and their actual knowledge about the use of MgSO4 including formal training.
In-depth interviews with pharmacists assessed procurement practices and stock-keeping for MgSO4 as well as the pharmacists’ knowledge on MgSO4 (dosage, preparation etc.) including formal training.
Research team planned to review the treatment charts of any admitted patient with severe pre-eclampsia and eclampsia at the time of visit.

Relevant information related to policies and regulations in vigour was extracted from the document review. Interviews and focus group discussions were audio-recorded and transcribed. Transcripts were analysed by two independent researchers and manually extracted data were categorized in a coding frame according to our framework (Fishbone Diagram). Qualitative data collected through observation at facility level were also analysed in the same way while quantitative data was entered in and analysed using statistical software (SPSS 15).

Data from all sources were combined to identify enablers and barriers to access to and use of MgSO4 by level of the health system. These results were presented to selected stakeholders from public and private health sector who gathered in a one-day round-table meeting in Islamabad, Pakistan in February 2011. Discussion and consensus building among stakeholders identified concrete actions and activities that would build upon existing facilitators and contribute to removal of health system barriers to access and use of MgSO4.

## Results

Barriers and enablers to the use of MgSO4 were assessed by level of the health system. The findings are detailed below.

### 1. Health Sector Level: Governance and Regulatory Issues

Magnesium sulfate injection is registered for use in Pakistan and licenced for both severe pre-eclampsia and eclampsia. It is listed as the first line treatment for eclampsia in key policy documents - NEML 2007 [13] and the training manuals for midwives [14]. Magnesium sulphate is however not a part of the 2009 Karachi Declaration on scaling up maternal, newborn, child health and family planning best practices [15]. There is no mention of the possibility of an intramuscular (i/m) only regimen in any of the policy documents.

Countrywide consumption data of MgSO4 in the public and private sector shows a rising trend in the utilization for 10 ml injections over the years. This usage however remains ten times lower than desired consumption based on incidence of pre-eclampsia and eclampsia.

### 2. Health Sector Level: Pharmaceutical Supply System

#### Production

Only one Pakistan based national pharmaceutical company (Zafa) is manufacturing the medicine and holds a de-facto monopoly on the market. Unlike other medications against eclampsia such as diazepam, which can be used for other indications as well, MgSO4 is only registered and effective for the treatment of severe pre-eclampsia and eclampsia, resulting in a small market for the medicine. Price of one 2 ml (500 mg/ml) ampoule varied from Rs 3 to 4 and is on average Rs 3.5 or US$ 0.04 per ampoule. Small market and low price combined significantly reduce the incentives for the pharmaceutical company to market the drug effectively and ensure adequate levels of production and national distribution.

#### Procurement and supply

Various methods for the procurement and supply of medicines are currently in place in Pakistan in the public and private sector and depending on the level of health facilities. Procurement of medicines for the public sector hospitals is carried out by the provinces and is based on the NEML, financed through the provincial health budget. The Provincial Medical Store Depot (MSD) manages the acquisition, storage and distribution of medicines for the province and is the main supplier of medicines to public sector hospital pharmacies and district health offices. Private hospitals acquire medicines from the open market. Central and specialized hospitals, such as the Pakistan Institute of Medical Sciences (a teaching hospital), are given a grant by the Ministry of Health for independent procurement of emergency medicines and medical supplies. These multiple procurement and distribution methods create discrepancies in the availability of all medicines throughout the country, which also affect MgSO4. The procurement of MgSO4 is mostly dependent upon the demand from the relevant hospital departments, which is not necessarily aligned with NEML and national policies. The combination of fragmented procurement and supply system and a lack of demand for the drug emanating from practitioners create a chronic situation where the drug is unavailable and underutilized.

### 3. Health Service Delivery Level: Health Facility

#### Availability of MgSO4

The availability of 24-hour supply of MgSO4 to treat one patient was assessed through interviews of pharmacy staff and on-site observation in all facilities visited. Table 1 reports on these results as well as availability of other medical supplies and equipment for correct administration of MgSO4 and the reasons reported for shortage of the drug. Magnesium sulfate was available in all teaching hospitals visited and only 20% of non-teaching hospitals. In 80% of non-teaching facilities visited, pharmacy staff indicated that procurement was dependent on demand from respective hospital wards. However, during the focus group discussions, obstetricians and other health personnel had concerns about availability of MgSO4 and indicated this was an obstacle to its use. None of the private hospital pharmacies surveyed did have the medicine in stock. Again, they reported that they only procured the drug if there was a demand from practitioners. The availability of necessary adjuncts medicines and supplies was usually good in teaching hospitals except for the antidote calcium gluconate which was missing in 2 out of 5 teaching facilities. Calcium gluconate was not available in any of the non-teaching hospitals. Local anesthetic lignocaïne for i/m regimen was only available in 60% of non-teaching facilities while the infusion pumps for i/v infusions were not available in any setting including in teaching hospitals.

**Table 1:** Availability of magnesium sulfate in health facilities.

	Teaching hospitals (n = 5)	Non-teaching hospitals (n = 10)
No of facilities which stock MgSO4 (pharmacy staff interviews)	5	0
No of facilities with sufficient quantity of MgSO4 to provide 24 h treatmentto one patient (on-site observation)	5	2
No of facilities with adequate supply of:		
Calcium gluconate	3	0
Lignocaïne	5	6
Sterile syringe	5	10
i/v pump	0	0
Main reason for the drug not being available (pharmacy staff interview)		
Lack of demand	NA	8
No profit or low cost	NA	1
Not registered	NA	0

#### Correct usage

The usage of MgSO4 At health facility level was assessed through interviews and is reported in Table 2. Magnesium sulfate was reported as a preferred first line treatment for eclampsia in all teaching hospitals and only in 3 non-teaching hospitals out of 10. For severe pre-eclampsia, only 2 of the 5 teaching hospitals and none of the non-teaching hospitals were using MgSO4. Interestingly, diazepam was not a preferred alternative, neither in teaching nor in non-teaching hospitals. In non-teaching hospitals, patients were referred to tertiary care in 50% of cases and therefore, MgSO4 was not used. None of the facilities were using neither i/m only nor i/v only regimen; the preferred route of administration remained i/v followed by i/m. The findings from the interviews and focus group discussions with the obstetricians unveil a large variation in the dosage quantities, which in most cases were not in line with the recommendations of international guidelines. During the discussions, health staff found the dosage preparation to be the biggest barrier to the use of the medicine as they have to recall, calculate and prepare the dosage themselves. Written protocols to guide local use of the drug could be found only in one non-teaching facility and in 4 teaching hospitals. In contradiction to official guidelines, diazepam ampoules were observed on the emergency trays in many sites although only 2 non-teaching hospitals report using it for eclampsia and none of the facilities report using it for severe pre-eclampsia. In a few places, presence of both diazepam and MgSO4 was observed and it should be noted that using these two medicines together is a harmful practice.

**Table 2:** Use of magnesium sulfate in health facilities.

	Teaching hospital (n = 5)	Non-teaching hospital (n = −10)
First treatment choice for eclampsia		
MgSO4	5	3
Diazepam	0	2
Referral	0	5
Other	0	0
First treatment choice for severe pre-eclampsia		
MgSO4	2	0
Diazepam	0	0
Referral	0	5
Other	3	2
No answer	0	3
Preferred route of administration		
i/m only	0	0
i/v only	0	0
i/v followed by i/m	5	3
Other	0	0
No of hospitals with a written protocol for MgSO4 administration	4	1
Who prepares the medicine for injection?		
Doctor	2	2
Nurse	3	0
Other (incl pharmacy)	0	0

#### Affordability

The price of MgSO4, on average at Rs 3.5 or US$ 0.04 per ampoule, is affordable for most patients. However the fragmented and disrupted procurement and supply mentioned by many stakeholders creates drug shortages and results in MgSO4 being sold at a much higher price in the open market (as high as Rs 75 or US$ 0.9 in Quetta). Furthermore, in four hospitals, it was reported that standard practice was to ask patients’ families to purchase the medicine and bring it to the hospital, a practice that is quite usual in the private sector.

### 4. Health Service Delivery Level: Health Professionals

Enablers and barriers to the use of MgSO4 at the level of health professionals may be related to the formal training that they have received or not, their knowledge, perceptions and the translation of these in their actual practices. These elements were assessed through interviews and focus group discussions.

#### Training

Staff attending pregnant women in 9 of the 10 non -teaching hospitals visited had not received any formal in-service training for the use of MgSO4. None of the pharmacy staff had received any training either. In teaching hospitals, postgraduate staff had received formal training as part of their pre-service education and nurses had been exposed to on-the job training, Informants and interviewees suggested that the training was sufficient in teaching hospitals, despite the incorrect practices that were noticed by researchers when discussing dosage and regimen with health staff and through on-site observation. The training was found to be an extremely facilitative factor for the use of MgSO4 even in the non-teaching facilities and it was observed that secondary care hospitals where some maternal care intervention had been implemented by donors were better equipped and were using MgSO4 injections for eclampsia.

Due to small numbers of eclampsia patients treated in some of the lower level facilities, a lack of recent knowledge and experience was reported, which is another potential barrier to effective administration of MgSO4. Discussants felt that training was not adequate in private settings.

#### Perception and Practices

One of the teaching hospitals visited had taken part in the initial clinical trials establishing efficacy and safety of MgSO4 (2, 3). Interviews and focus groups showed that health professionals were mostly aware of the usefulness of MgSO4 as the first line treatment for eclampsia, though many had no knowledge of its use in severe pre-eclampsia. Safety issues were reported as motivating the choice of first line treatments other than MgSO4 (i.e. diazepam or referral). There was a “fear” of using MgSO4 among the health professionals; the staff in many of secondary care hospital and basic health units immediately referred convulsing patients without any emergency management as they felt inadequate and there were no referral guidelines available for use. Anecdotal evidence during discussions showed that the relatives would sometimes take the woman directly to a tertiary care even if it was far off, leading to repeated convulsions and higher risk of mortality. There was a general misconception that this drug can only be used in advanced settings with intensive care services. When asked whether primary health care should be using MgSO4, respondents and focus group participants agreed that it can benefit women but stressed the need for proper training and availability of packaged and ready-to-use dilutions. There were concerns of MgSO4 toxicity especially among the older generation of physicians. The lack of adequate level of bedside nursing allowing adequate monitoring of MgSO4 administration was reported as a bottleneck. An obstetrician reported: “I lost one patient because the i/v infusion was turned faster by a relative, so I will not use it as it is not safe without monitoring”.

### 5. Summary of Barriers and Enablers

Factors that enable access and use of MgSO4 are summarized in Table 3 and reflect the adequate translation of clinical evidence on the effectiveness of the drug into national policies. These mainly consist in the fact that the drug is registered in Pakistan and licensed for its indications, listed in NEML and main policy documents, although more efforts are needed to list it in all relevant guidelines at national level. A local manufacturer produces the medicine at a low price and the country does not need to rely on international supply. Theoretically, procurement is based on NEML. Demand from practitioners exists, there are facilities that use MgSO4 for both indications and can be used as model facilities for awareness raising and training. National champions can be found among obstetricians, especially as Pakistan was among the countries participating in the original Magpie collaborative trials that demonstrated the effectiveness of the drug (2). Many health staff have received pre- or in-service training and are aware of the usefulness of the drug for treatment of eclampsia. They are also cautious of its potential adverse effects.

**Table 3:** Enablers and barriers to access and use of magnesium sulfate in Pakistan.

Health system level	Enablers	Barriers
**Health sector level**		
**Governance and regulatory** **issues**	MgSO4 registered in Pakistan. MgSO4 licensed for bothindications of eclampsia and pre-eclampsia. MgSO4 listedin NEML and MNCH EmONC training manual.	MgSO4 not part of Pakistan Best Practices Policy. No specific guideline for MgSO4 use. National policy documents do not mention i/m route of administration.
**Pharmaceutical supply system**	Public hospitals procurement theoretically based on NEMLwhere MgSO4 is listed.	Fragmented procurement system for different types of facilities. Procurement in practice depends on demand from hospital wards.
	Local pharmaceutical company manufactures MgSO4.Generic form available. Low price.	De-facto market monopoly of one single pharmaceutical company. Single indication, small market and low price are a disincentive for marketing strategies. Higher prices encountered in private markets due to disrupted procurement.
**Health service delivery level**		
**Health facilities**	Demand from hospital wards exists. Patient referred tofacilities where MgSO4 is in use. Model practiceexists in selected places.	MgSO4 unavailable in adequate supply.Diazepam still in use. Referral of patients without stabilization, referral guidelines unavailable. Adjunct drugs and medical supplies unavailable in adequate supply. Large variations in dosage and regimen practiced. Absence of local written protocol. Preparation of injections by doctors, nurses or midwives.
**Health professionals**	National champions exist. Some health staff has received training related to MgSO4. Health professional aware ofMgSO4 usefulness in treatment of eclampsia.Health professionals cautious of safety and adverse effectsof MgSO4.	Pharmacists in particular have received no training related to MgSO4. Refresher training programs and educational reminders not in place for lower levels of care. Health professionals unaware of MgSO4 usefulness in treatment of severe pre-eclampsia. Misconceptions and negative experiences on safety and toxicity of MgSO4. Weak level of bedside nursing, weak monitoring of MgSO4 administration.

Barriers to access to and use of MgSO4 are however more complex and outweigh existing enablers (see Table 3): they reflect the lack of adequate translation of national policies into implementation arrangements. They are related to fragmentation of procurement and supply based on demand from practitioners rather than on NEML or national policies. De-facto monopoly of a single pharmaceutical company and a small market creates disincentive for marketing the drug. Persisting practices of using diazepam or immediate referral without stabilization also act as bottlenecks. There are no local protocols nor referral guidelines, and therefore little support for health staff to use MgSO4. Refresher training and education reminders are needed for staffs who believe that the drug is unsafe to use outside facilities with intensive care.

These enablers and barriers were presented to a group of national stakeholders during a one-day round-table meeting in Islamabad, Pakistan in February 2011. The group validated the findings of the study and discussed the range of actions that could be taken to overcome existing barriers. They built this action plan on evidence collected during this study and existing enablers that could be used to create positive synergies (see Table 4).

**Table 4:** Action plan for access to and use of magnesium sulfate in Pakistan.

Health system level	Actions required
**Health sector level**	
	**Government/regulatory**
	Raise awareness.
	Include disease and MgSO4 in national priorities and plans.
	Prepare standard national treatment guidelines for MgSO4 use.
	Ensure coherence with NEML and other STG in place.
	**Pharmaceutical supply system**
	Allocate earmarked budget for procurement of MgSO4.
	Facilitate flow of information for adequate forecast of MgSO4 stock.
	Negotiate with pharmaceutical company for production and distribution of required quantity of MgSO4.
	Explore registration and licensing of pre-diluted dosage forms.
	Explore registration and licensing of MgSO4 injection kits.
**Health service delivery level**	
	**Health Facilities**
	Monitor availability at facility level. Facilitate formal communication between hospital departments for adequate forecast of necessary stock.
	Translate national policies and guidelines into simplified standard protocols.
	Design simple and visual treatment aid in local languages and disseminate in all facilities.
	Monitor use of protocols and treatment aids.
	Prepare and disseminate referral guidelines.
	Consult with facilities for new formulations.
	Promote/reward model health facilities/model obstetric wards, encourage facilities or wards to become a model.
	Health Professionals
	Implement in-service training in all facilities. Disseminate treatment aid and monitor its use by health professionals
	Follow-up with regular refresher trainings, peer-to-peer training and coaching.
	Collect feedbacks on treatment aid and adapt to specific needs
	Consult with health professionals on new formulations
	Promote champions, use them for peer-coaching
	Develop pre-service training

Eclampsia and severe pre-eclampsia must be placed high on the priorities and plans at national level. National standard treatment guidelines should be prepared for MgSO4, aligned with NEML and other STG in place; translated in local protocols and on-the job treatment aids in local languages. The use of these protocols and treatment aids should be monitored at facility level; feedback from health staff should be collected and used to adapt the protocols and treatment aids to specific needs. An earmarked budget for procurement of MgSO4 must be defined and available for different procurement channels. Communication and information sharing should be facilitated between hospital wards, pharmacy units and procurement agencies to allow procurement based on required supply of the drug. Health authorities must engage in negotiations with the pharmaceutical company to ensure adequate levels of production and distribution of MgSO4, whereby correcting the market failure with alternate incentives for the company, such as a secure and stable consumption. Feasibility and cost-effectiveness of producing pre-diluted formulations which would not require preparation by health staff in obstetric wards should be explored, as well as possibility of pre-packaged injections kits combining MgSO4 ampoules, sterile injection supplies, calcium gluconate and lignocaine. Facilities and health staff must be consulted on these new formulations; registration and licensing should be further facilitated. Availability and use of the drug at facility level should be monitored and the information fed-back to health staff, pharmacy departments and procurement agencies. In-service training should be expanded to all staff and facilities dealing with eclampsia and severe pre-eclampsia; facilities and staff that already use the drug may be rewarded and promoted as models and champions, used in peer-training and coaching. Refresher training is also necessary as well as simple and fast educational reminders. Pre-service training should be aligned with national guidelines, standard protocols and in-service training.

Monitoring indicators are necessary to evaluate progress made against the action plan. These indicators should be defined nationally and translated into local level indicators; baseline status should be assessed and targets should be set with defined timelines. Such indicators could include (but would not be limited to) an increase in MgSO4 production, availability levels in public health facilities, actual usage of correct dose and regimen, number of trained staff by level of care.

In the long term, efforts should also engage the private health service provisions. All recommendations of the action plan, including monitoring indicators are relevant for private health facilities although it is recognized that their implementation and enforcement in the private sector are a challenge in a fragile and highly pluralistic health system.

The action plan must be translated in a specific set of activities through consultation with the relevant group of stakeholders. Agencies or authorities which should take the lead in the implementation of the action plan should be clearly defined and given an adequate mandate to perform the work. Finally, the action plan should be costed and national as well as donors’ resources should be identified and earmarked to support its implementation.

## Discussion

Lumbiganon *et al*. [8] have formally assessed underutilization of MgSO4 in Mexico and Thailand; they suggested several explanations such as failure in registration, distribution and procurement, the low price of the drug, and reluctance of staff to use it because of required intensive monitoring. These barriers have been confirmed by Sevene *et al*. [9] in Mozambique and Zimbabwe; and Ridge *et al*. [10] in Zambia. We found that the situation in Pakistan was similar to the above mentioned countries although several enablers exist in Pakistan that were not present in other countries, especially registration of MgSO4 and its licensing for both indications of pre-eclampsia and eclampsia. Aaserud *et al*. [16] suggest a few additional barriers, identified through discussions and interviews of the original Magpie trial collaborators [2]: training of health staff on the use of MgSO4, translation of guidelines into local protocols and active implementation of these. Our study confirms these findings. Similarly to Ridge *et al*. [10] we found that evidence on the effectiveness of the drug has been successfully translated into policy in Pakistan, while translation of policy to practice has remained weak and is the main determinant of low access and use.

The strength of our study is the adoption of a health system perspective on both enablers and barriers to access and use of MgSO4, using mixed methods and a comprehensive data collection approach. Ridge *et al* [10] have only observed a pragmatic sample of three facilities and recognized that qualitative methods would have been useful to assess knowledge and experience of staff at health facilities. Sevene *et al* [9] have performed interviews with a limited number of clinicians, researchers and policy makers but did not perform on-site observation at facility level to assess health service delivery bottlenecks. In our study, policy document review and on-site observations were combined with semi-structured interviews and focus group discussions with a wide range of stakeholders including facility level clinical and pharmacy staff, policy and decision makers, opinion leaders, researchers and lecturers: this approach has brought depth in the understanding of context-specific bottlenecks to access and use of this life-saving medicine. While adopting the health system perspective put forward by Ridge *et al* [10] we have strengthened the methodological basis of this work through broader data collection and analysis and inclusion of a comprehensive range of stakeholders.

Bigdeli *et al* [12] suggest that system responses to interventions aimed at improving access to medicines should be adequately analyzed at all levels of the health system (international and national above the health sector, health sector, and health service delivery and population levels). These system responses, whether positive or negative should be used in re-design of interventions in order to create positive feedback loops for a better access. Our study suggests that registration and licensing of MgSO4, local production and distribution, have not been effective in ensuring access and use of the drug for women with severe pre-eclampsia and eclampsia. We have found that these actions have taken place only at one level of the health system, namely the health sector level (focusing on regulatory aspects and procurement), while extensions of the intervention at the service delivery level have been neglected in Pakistan. The benefit of our study is to bring new context-specific insight into operational and service delivery experiences in using MgSO4, an approach that is advised in the emerging field of Health Policy and System Research [17], [18].

The most important innovation of our research is the formulation of an action plan, resulting from the presentation of our research findings to a round-table of stakeholders and further debate and consensus-building. This has supported an evidence-based policy dialogue. Indeed, Gilson *et al* [18] argue that a challenge of Health Policy and System Research is to initiate public debate around research findings and whereby support policy and system change. According to Paina and Peters [19], successful scaling-up of health interventions require understanding the dynamic internal model of health care practices, identifying critical leverage points and using them to bring about change. In addition to an in-depth understanding of practices related to MgSO4, our study has offered the opportunity of sharing this knowledge with decision makers and advocating for practical actions that would trigger change. Stakeholders engaged in the round-table discussion in Pakistan have acknowledged the existence of enablers to access and use of MgSO4 such as the regulatory environment, local production and low cost; they have also recognized that there are champions of the case such as obstetricians involved in the original Magpie collaborative trial [2], facilities and practitioners who administer the drug routinely and who can be used as role models. These enablers are critical leverage points based on which the action plan was formulated.

Our study has several limitations. Firstly, it does not explore in detail three important levels of the health system namely the population level, national level beyond the health sector and the international level, where barriers to access and use of MgSO4 may occur [12]. We have touched briefly upon the fact that MgSO4 is a cheap medicine available in generic form, recognized internationally as the first line treatment of eclampsia and severe pre-eclampsia and pointed by various international organizations as an essential commodity, necessary to attainment of MDG 5. We therefore believe that barriers at international level are limited and may not hamper access and use. At population level, barriers are related to access to antenatal care, early diagnosis of pre-eclampsia and eclampsia and follow-up during delivery. These are important barriers that however affect access to any treatment of pre-eclampsia and eclampsia - MgSO4 or other - and do not specifically influence low access and use of MgSO4 compared to alternatives such as diazepam. Second, the policy dialogue and formulation of the action plan, although based on locally relevant and context-specific evidence, adopted a typical “research push” approach [20], whereby researchers present their findings to policy makers and advocate for policy change. It has been argued that such approach is not the most effective knowledge brokering method [20] and that effective translation of research into policy and practice requires institutionalization and an interactive “push-pull” method, with constant dialogue and exchange between researchers and policy makers. The Health Services Academy in Islamabad is however close to central level policy makers and in a position to interact effectively with key stakeholders for an effective implementation of the action plan. An active follow-up is now needed, with seed resources that should be used to implement the next steps suggested during the round table meeting: translation of the action plan into a specific set of activities, identification of agencies that will take the lead in the implementation of various elements of the action plan, with a clear mandate and earmarked resources to perform the work.

### Conclusion

This study highlights the complexity of ensuring adequate access to cheap and life-saving essential medicines in LMIC. It is yet another illustration that a vertical view of regulatory and procurement requirements is not sufficient to address the multiple barriers to access and use. It demonstrates the need to embed access to medicines within a health system perspective and identify enablers and barriers at multiple levels of the health system, especially at operational and health service delivery levels.
